# Prevalence and determinants of antenatal depression in Ethiopia: A systematic review and meta-analysis

**DOI:** 10.1371/journal.pone.0211764

**Published:** 2019-02-19

**Authors:** Getinet Ayano, Getachew Tesfaw, Shegaye Shumet

**Affiliations:** 1 Research and Training Department, Amanuel Mental Specialized Hospital, Addis Ababa, Ethiopia; 2 Department of Psychiatry, University of Gondar, Gondar, Ethiopia; Erasmus Medical Center, NETHERLANDS

## Abstract

**Background:**

Maternal depression is the most prevalent psychiatric disorder during pregnancy, can alter fetal development and have a lasting impact on the offspring's neurological and behavioral development. However, no review has been conducted to report the consolidated magnitude of antenatal depression (AND) in Ethiopia. Therefore, this review aimed to systematically summarize the existing evidence on the epidemiology of AND in Ethiopia.

**Methods:**

Using PRISMA guideline, we systematically reviewed and meta-analyzed studies that examined the prevalence and associated factors of AND from three electronic databases (PubMed, EMBASE, and SCOPUS). We used predefined inclusion criteria to screen identified studies. A qualitative and quantitative analysis was employed. Heterogeneity across the studies was evaluated using Q and the I² test. Publication bias was assessed by funnel plot and Egger’s regression test.

**Results:**

In this review, a total of 193 studies were initially identified and evaluated. Of these, five eligible articles were included in the final analysis. In our meta-analysis, the pooled prevalence of AND in Ethiopia was 21.28% (95% CI; 15.96–27.78). The prevalence of AND was highest in the third trimester of pregnancy at 32.10% and it was 19.13% in the first trimester and 18.86% in the second trimester of pregnancy. The prevalence of AND was 26.48% and 18.28% as measured by Beck depression inventory (BDI) and the Edinburgh Postnatal Depression Scale (EPDS), respectively. Moreover, the prevalence of AND was 15.50% for the studies conducted in the community setting and it was 25.77% for the studies conducted in the institution-based setting. In our qualitative synthesis, we found that those pregnant women who had a history of stillbirth, complications during pregnancy, previous history of depression, no ANC follow-up, irregular ANC follow-up, not satisfied by ANC follow-up, and monthly income <1500 Ethiopian birr were linked with a greater risk of developing ANC. We also found that those women who experienced partner violence during pregnancy, food insecurity, medium and low social support, and those who were unmarried, age group 20–29, house wives and farmers were associated with a higher risk of developing ANC.

**Conclusion and recommendations:**

Our meta-analysis found that the pooled prevalence of AND in Ethiopia was 21.28%. The prevalence of AND was high in the third trimester of pregnancy as compared to the first and second trimesters of pregnancy. The prevalence of AND was high in studies conducted using BDI than EPDS. Studies on the magnitude of AND as well as the possible determinants in each trimester of pregnancy with representative sample size are recommended. Screening of depression in a pregnant woman in perinatal setting might be considered backed by integration of family planning and mental health services. The use of validated and a standard instrument to assess AND is warranted.

**Systematic review registration:**

The protocol for this systematic review and meta-analysis was registered at PROSPERO (record ID=CRD42017076521, 06 December 2017)

## Background

Pregnancy is the period of great joy and positive expectations, but also physical and mental stress and difficulties. Pregnancy is associated with a range of physiological as well as psychological changes for the mothers and they are expected to face numerous new challenges in this period. As a result, the perinatal period is associated with a considerably greater risk of experiencing mental health problems for all women [1, 2]. Of the varies mental health problems, maternal depression is the most prevalent psychiatric disorder during pregnancy [1, 3, 4], can alter fetal development and have a lasting impact on the offspring’s neurological and behavioral development [5, 6].

Epidemiologic studies showed that the estimated prevalence of antenatal depression (AND) varies between studies. The estimated prevalence of AND varies between 7% and 20% in developed countries depending on the study [7–10]. According to a recent meta-analysis, the pooled prevalence estimates of antenatal natal depression (AND) in the low and middle-income countries (LMICs) was 25.3% (95% CI 21.4–29.6%) [11], whereas, the reported prevalence estimates of AND ranges between 4% and 56% [1, 3, 5, 12–22]. The type of the instrument used to assess depression, the study setting, the trimester of pregnancy, the socioeconomic and cultural differences are among the common possible reasons for the observed variation in the magnitude of AND [1, 3, 5, 12–22].

Scientific studies also found that AND commonly precedes postnatal depression [23] and associated with a higher risk of suffering to the woman as well as her family[24]. In addition, it has been shown that AND is among the leading causes of perinatal morbidity and mortality in women [25].

The evidence from the reported prevalence estimate of AND shows that the prevalence of AND was high in LMICs, such as, 20.2 % in Brazil [26], 25 % in Pakistan[27], 29 % in Bangladesh [28], 39 % in South Africa Cape Town, 38.5 %[29] in South Africa KwaZulu-Natal[30] and 39.5 %, in Tanzania[31]. The estimated prevalence of AND in Ethiopia ranges between 11.8% and 31.1% depending on the study [32–36].

Moreover, the estimated prevalence of AND varies depending on the different trimesters of pregnancy. For example, according to the results of a meta-analysis conducted in the LMICs, the magnitude of AND was 7.4 % (2.2–12.6 %) in the first trimester, and it was 12.8 % (10.7–14.8 %) in the second trimester and 12.0 % (7.4–16.7 %) in the third trimester of pregnancy [1].

Epidemiological studies has identified several factors that were responsible for an increased risk of AND such as, younger age, unplanned pregnancy, unmarried by marital state, lack of family support, lack of spouse support, past history of psychiatric disorders, violence during pregnancy, low socioeconomic status, stressful life events, first pregnancy, poor antenatal care follow up, pregnancy-related complications and previous operative delivery [32–37].

Even though we found a remarkable variation in the magnitude of AND among the studies conducted in Ethiopia in addition to the observed methodological weakness associated to the measurement and substantial inconsistency across the findings, there are no previous systematic review and meta-analysis studies conducted in Ethiopia on the prevalence and determinates of AND. Therefore, the objective of this study was to perform a systematic review of studies conducted in Ethiopia on prevalence and predisposing factors of AND and to systematically summarize: (1) the prevalence of AND (2) the prevalence of AND during the three trimesters of pregnancy (3) the determinants of AND, and to formulate recommendations for future research.

## Methods/design

This systematic review and meta-analysis was conducted following “the Preferred Reporting Items for Systematic Reviews and Meta-Analyses (PRISMA)” guidelines [38]. We conducted an extensive search of the literature in three databases (EMBASE, MEDLINE, and Scopus). The following terms and keywords were applied for PubMed search: (prevalence OR magnitude) AND (Antenatal OR Pregnancy OR prenatal OR women) AND (Depression OR depressive symptoms OR depressive disorder OR depressive) AND (Ethiopia). For the other two electronic databases (EMBASE and SCOPUS) we used database specific subject headings linked with the above terms and keywords used in PubMed. We also screened at the reference lists of the remaining papers to identify additional relevant studies to this review. This review protocol was written and presented according to PRISMA-P 2015 guidelines [39].

### Systematic review registration

The protocol for this systematic review and meta-analysis was registered at PROSPERO International Prospective Register of Systematic Reviews 2017 (record ID=CRD42017076521, 06 December 2017)

#### Definition of concepts

In this review, institution base study refers to the study conducted in a clinical setting such as hospital and health centers whereas the community-based study refers to the study conducted in non-clinical settings including household surveys or home interviews.

### Eligibility criteria

Two investigators (GA and GT) independently screened the selected studies using their titles and abstracts before retrieval of full-text papers. We used prespecified inclusion criteria to further screen the full-text articles. Disagreements were discussed during a consensus meeting with a third reviewer (SS) for final selection of studies to be included in the systematic review and meta-analysis.

### Inclusion criteria

- Design type—observational studies
- Study participants–pregnant women
- Those articles published in the English language
- Studies that reported on either prevalence and/or determinants of antenatal depression
- Studies were done in Ethiopia

### Exclusion criteria

- Studies dealing with depressive disorders in nonpregnant women
- Studies dealing with perinatal depressive disorders associated with the postpartum period
- We also excluded editorials, letters, reviews, commentaries, and interventional studies
- Studies whose full data was not accessible even after requests from the authors were also excluded
- We also excluded duplicate studies

### Data extraction

Two investigators (GA and GT) independently extracted the data from the studies included in our analysis as recommended by PRISMA guidelines [39]. The data was extracted using a standard data extraction form. The following information was extracted from the selected studies: first author’s name, study population, the type of study design, sample size, study setting, data collection instrument, year of publication, tools used for assessing outcome, measures of effect (odds ratio (OR)), confounding variables, and the possible associated factors results.

### Data synthesis and quality assessment

In this study, all the analysis was conducted using comprehensive meta-analysis software version3 [40]. We pooled the overall prevalence estimates of AND by a random effect meta-analysis [41]. We examined the heterogeneity of effect size using Q statistic and the I² statistics[41]. The Q-test measures whether the observed effect size is considerably different from one another than expected by chance. When Q test is higher than the degree of freedom it indicates significant heterogeneity (also supplemented by P value). The I² statistics assess the proportion of total variance across the included studies contributed to the observed heterogeneity. In this study, the I^*2*^ statistic value of zero indicates true homogeneity, whereas the value 25, 50 and 75% were considered to represent true, low, medium and high, respectively [42]. For the data identified as heterogeneous, we conducted our analysis by random-effects model analysis. When statistical pooling is not possible, non-pooled data was presented in table form.

We evaluated the quality of selected studies by a modified version of the Newcastle-Ottawa Scale (NOS) [43]. As recommended by NOS scale, we evaluated the included studies by the following domains: first, sample size and representativeness; second, comparability between participants; third, ascertainment of antenatal depressive symptoms, and statistical quality. To evaluate the agreement between the two reviewers we used actual agreement and agreement beyond chance (unweighted Kappa).

We also conducted a leave one- out sensitivity analysis to appraise the main studies that exert a most important impact on between-study heterogeneity.

Moreover, to further identify the possible source of heterogeneity among the studies we conducted subgroup and sensitivity analysis based on the stages of pregnancy, the instrument used to measure AND, the study setting, and the quality of the included studies.

Finally, the funnel plot and Egger's regression tests were used to measure the presence of substantial publication bias.

## Results

### Identification of studies

**Fig 1:**
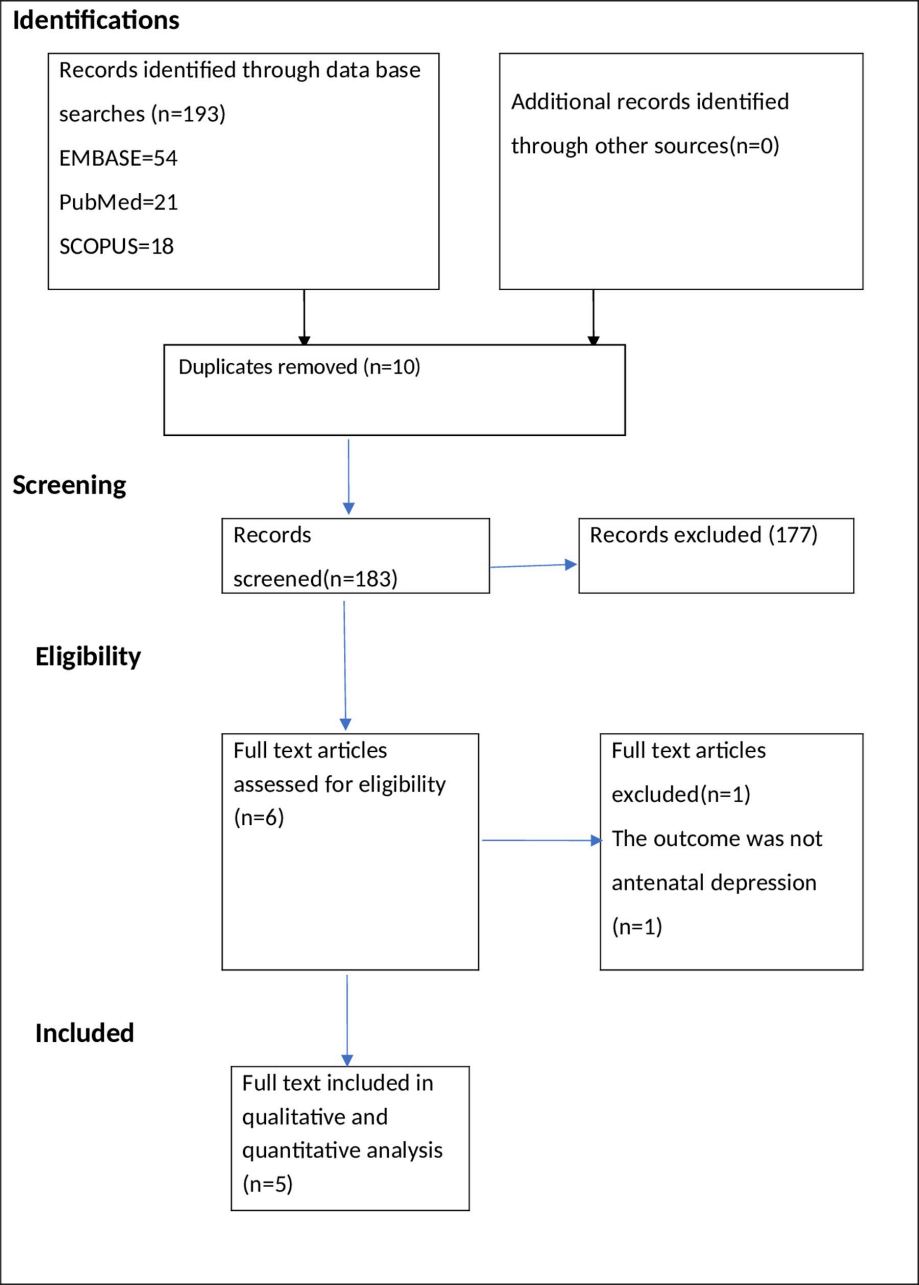
PRISMA flowchart of review search.

A total of 193 studies were identified using electronic search engine and strategies. Of these, 186 were excluded during the evaluation of duplicate and titles as they did not meet the inclusion criteria **(Fig 1).** The evaluation of abstract resulted in the exclusion of a further one article. Therefore, a full-text of 6 studies were retrieved for further assessment and one of these was excluded.

### Characteristics of included studies

Five papers were included in this systematic review and meta-analysis [32–36]. The characteristics of the included articles were shown in Table 1. Selected studies were conducted between 2013 and 2017. All studies included in the final analysis used a cross-sectional study design. Two of the studies used community samples [32, 36] and three of the studies used samples from the institution [33–35]. Regarding the instrument used measure AND, two of the studies used beck depression inventory (BDI) [33, 34] whereas three of the studies used Edinburgh Postnatal Depression Scale (EPDS) [32, 35, 36] to measure antenatal depression (**Table 1).**

**Table 1:** Distribution of studies on antenatal depression included in the qualitative analysis based on year, study design, setting, sample size, instrument, and prevalence.

Author(year) (reference number)	Study design	Setting	Sample size	Tool	Prevalence	response rate	Sampling
**Bisetegn TA. et.al (2016) [29]**	Cross sectional study	Community based	527	EPDS	11.8%	97%	Cluster sampling
**Ayele TA et. al. (2016) [30]**	Cross sectional study	Institution based	388	BDI	23%	92.82%	Systematic random sampling
**Biratu, A.,et. al (2015) [31]**	Cross sectional study	Institution-based study	393	EPDS	24.94	93.13%	Random sampling
**Dibaba Y et.al (2013) [33]**	Cross sectional study	Community-based study	622	EPDS	19.99%	99%	Census (as base line assessment for community-based cohort study)
**Mossie TB.et.al (2015) [32]**	Cross sectional study	Institution based	196	BDI	31.1%	93.8%	Systematic random sampling

Key: BDI: Beck depression inventory, EPDS: Edinburgh Postnatal Depression Scale

### Quality of included studies

In our quality evaluation, we found that all the included studies were of reputable methodological quality (NOS) score ranges between 8 to 9 from a total 9-point NOS score). Investigators reached an agreement that the risk of selection, ascertainment and non-response bias was low. A moderate or almost perfect agreement was found between investigators regarding the level of bias for the studies included in the final analysis (Kappa statistic range 0.50–1 (**S2 Table**).

### The results of a pooled meta-analysis

#### Prevalence of AND

**Fig 2:**
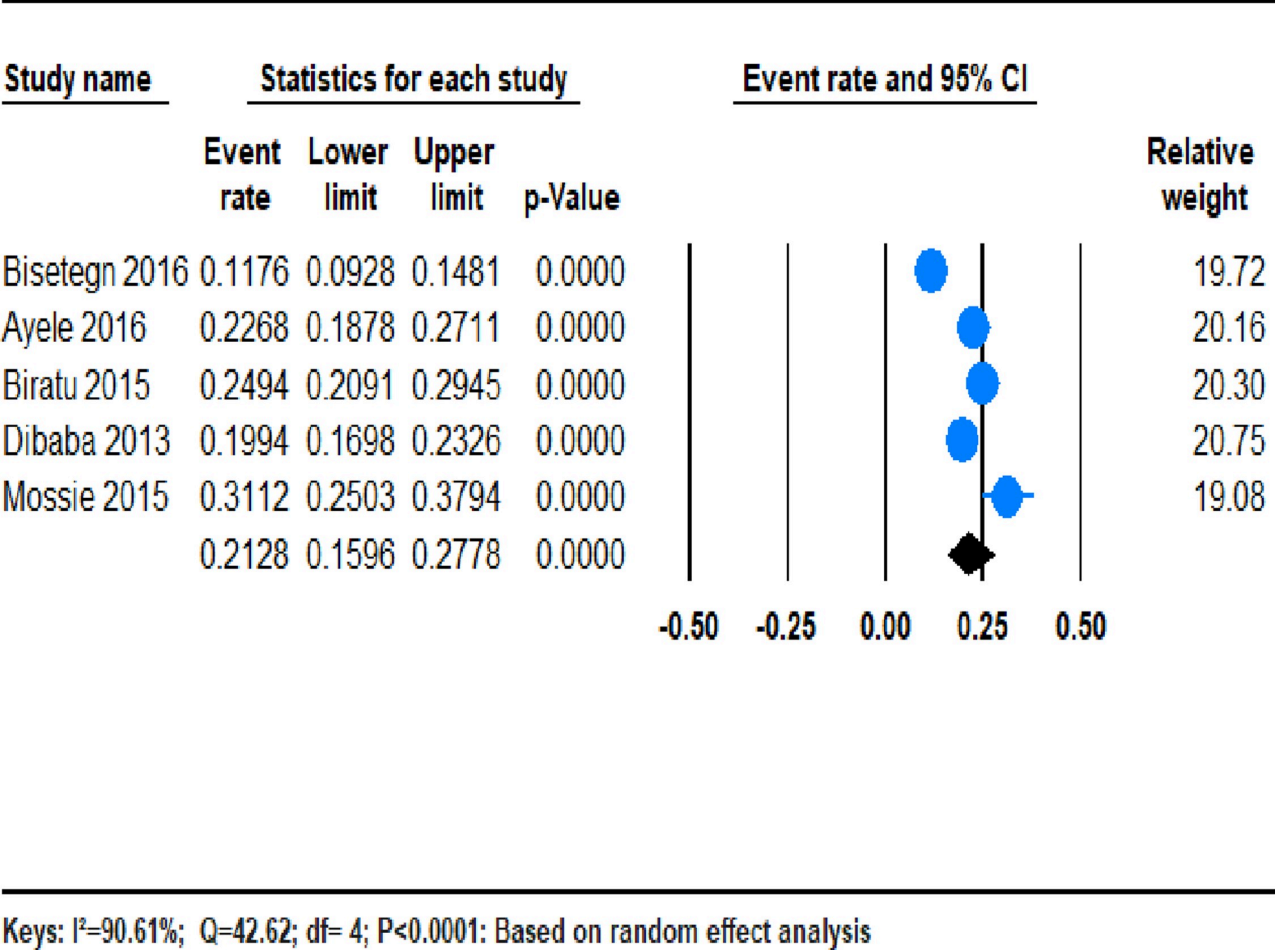
**The prevalence of AND in Ethiopia:** a meta-analysis.

Five studies were included in the meta-analysis, resulting in a reasonable samples size [32–36] **(Table 1).** From those studies, the pooled prevalence of AND in Ethiopia was 21.28% (95% CI;15.96–27.78). We found a significant heterogeneity among the five studies (*I*^2^ = 90.61%; Q = 42.61, df = 4, variance = 0.014, p <0.001)**. (Fig 2).**

#### The subgroup analyses prevalence of AND during the three trimesters of pregnancy

We conducted a subgroup analysis of studies which provided information regarding the prevalence of AND in each trimester of pregnancy. Of the totally included studies, four studies measured the prevalence of AND in the second and third trimesters of pregnancy, whereas only two studies estimated the prevalence of AND in the first trimester of pregnancy. The results of the subgroup analysis showed that the pooled prevalence of AND was highest in the third trimester of pregnancy at 32.10% (95%CI;12.93%, 60.07%), and it was 19.13% 19.13% (4.16%, 56.31%**)** in the first trimester and 18.86% (95%CI;11.51%, 28.87%) in the second trimester of pregnancy.

We found a considerable heterogeneity (*I*^2^ = 88.84%; p = 0.003), (*I*^2^ = 85.86%; p<0.001), and (*I*^2^ = 98.22%; p <0.001) for the first, second, and trimesters of pregnancy respectively (See **Table 2).**

**Table 2:** Subgroup analysis of the prevalence of antenatal depression in Ethiopia based on random effect analysis.

Subgroup	Number of studies	Estimates		Heterogeneity across the studies		Heterogeneity between groups (*P* value
		Prevalence (%)	95% Confidence interval	I^2^ (%)	P value	
**Trimester of pregnancy**						
First	2	19.13	4.16–56.31	88.84	P = 003	0.544
Second	4	18.86	11.51–28.87	85.86	P<001	
Third	4	32.10	12.93–60.07	98.22	P<0001	
**Instrument use**						
BDI	2	26.48%	19.09–35.48	79.38	P = 0.028	P = 0.032
EPDS	3	18.82	12.13–26.60	92.51	P<001	
**Setting**						
Community based	2	15.50	9.05–25.27	92.73	P<001	0.056
Institution based	3	25.78	21.62–30.41	59.39	P = 0.085	
**NOS scale quality score**						
Above 8	2	16.50	14.50–18.80	92.49	P<001	0.051
Less than or equal to 8	3	25.40	22.80–28.20	59.34	P = 0.085	

#### Subgroup analysis of the prevalence of AND by the instrument used

We also conducted a subgroup analysis by the type of instrument used to measure AND. The pooled prevalence of AND was 26.48% (95%CI; 19.09%, 35.48%) and 18.28% (95% CI;12.13–26.60) for studies conducted by using BDI and EPDS respectively. The heterogeneity was significant for both studies performed by BDI (*I*^2^ = 79.38%, Q = 4.85, df = 1, p = 0.028) as well as EPDS (*I*^2^ = 92.51%, Q = 26.72, df = 2 p<0.001). (See **Table 2).**

#### Subgroup analysis of the prevalence of AND by study setting

Finally, we conducted a subgroup analysis based on the study setting. The pooled prevalence estimates of AND was 15.50% % (95% CI;9.05–25. 27%) for the studies conducted in the community setting and it was 25.77% (95% CI 21.62–30.41) for the studies conducted in the institution-based setting. The heterogeneity was significant for community setting *I*^2^ = 92.73%, Q = 13.759, df = 1, p<0.0001) as well as the institution-based setting (*I*^2^ = 59.39%, Q = 4.921, df = 2, p = 0.085). (See **Table 2).**

### Publication bias

**Fig 3:**
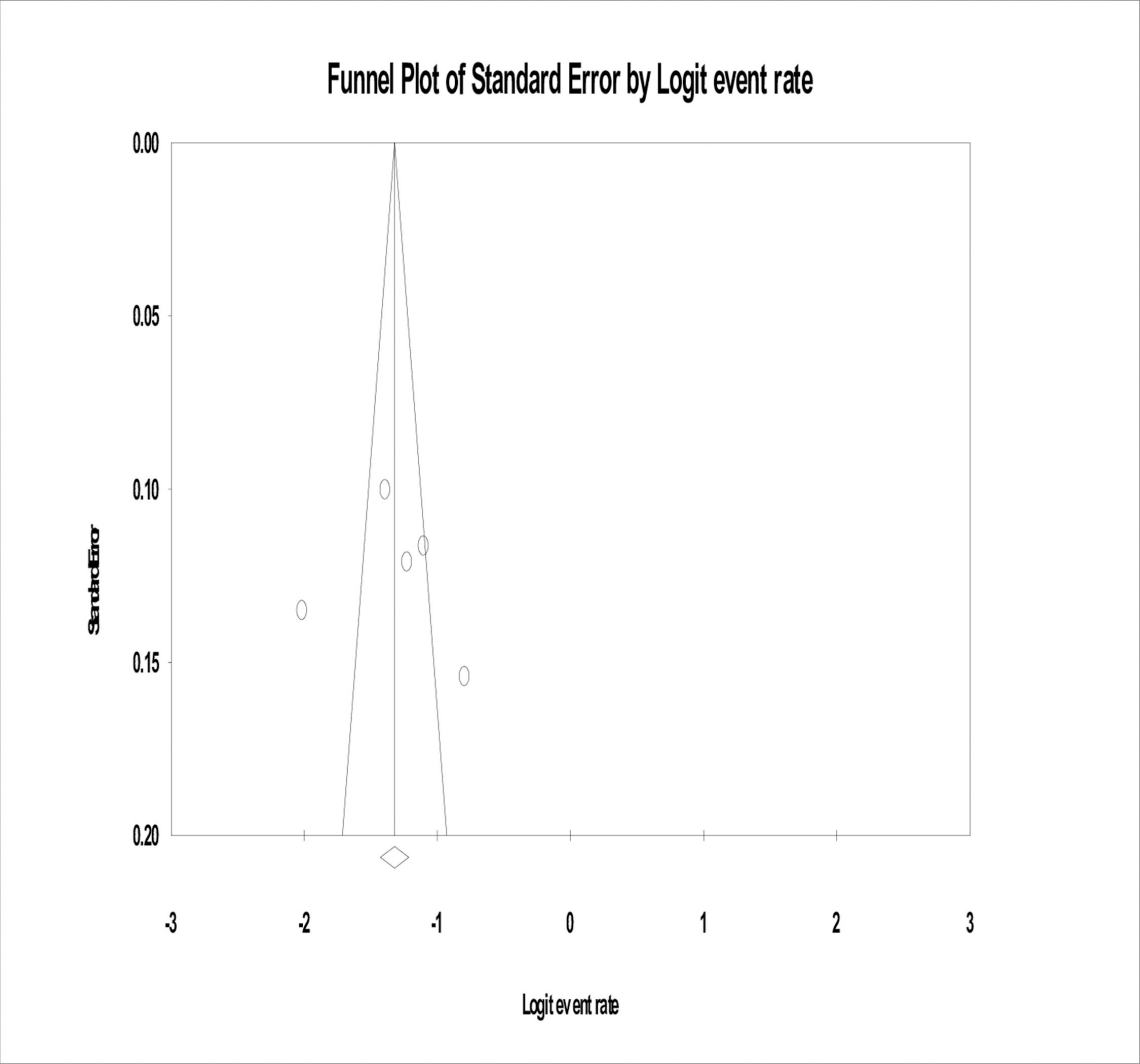
Funnel plot of risk of publication bias for the prevalence of antenatal depression in Ethiopia.

In the present study, no evidence of substantial publication bias was provided by the funnel plot and Egger’s regression tests (B = 2.66, SE = 11.75, P = 0.84) for the prevalence of AND in Ethiopia (**Fig 3**).

### Sensitivity analysis

We employed a leave-one-out sensitivity analysis to identify the potential source of heterogeneity in the analysis of the prevalence of AND in Ethiopia. The results of this sensitivity analysis showed that our findings were robust and not dependent on a single study. Our pooled estimated prevalence of AND varied between 19.93 (14.21–20.17) and 24.12 (25.5–30.84) after deletion of a single study **(see Table 3).**

**Table 3:** **Sensitivity analysis of prevalence for each study being removed at a time:** Prevalence and 95% confidence interval of antenatal depression in Ethiopia.

Study excluded	prevalence	95%CI
**Bisetegn TA. et.al (2016) [32]**	24.12	20.17–28.56
**Ayele TA et. al. (2016) [33]**	20.95	14.40–29.45
**Biratu, A.,et. al (2015) [35]**	20.42	14.21–28.46
**Dibaba Y et.al (2013) [36]**	21.65	14.62–30.84
**Mossie TB.et.al (2015) [34]**	19.33	14.33–25.56

Key. The analysis is based on the random effect model

To identify the source of heterogeneity we further conducted sensitivity analysis by study setting, the instrument used to ascertain AND as well as the prevalence of AND in each trimester of pregnancy. When limiting our analysis to the prevalence of AND in each trimester of pregnancy, the prevalence of AND was highest in the third trimester of pregnancy (32.10), as compared to the prevalence estimate in the first (19.13%) and second (18.86%) trimesters of pregnancy, although the difference was not statistically significant (P = 0.544). (See Table 2).

Moreover, when restricting our analysis to the prevalence of AND measured by using specific tools used to measure AND, the prevalence was highest in studies conducted using BDI (26.48%) than studies conducted using EPDS (18.82%). We found significant heterogeneity between the studies conducted using BDI and EPDs with a significant p-value between the group variations (P = 0.032). (See Table 2).

Additionally, in our stratified analysis by the study setting, the prevalence of AND was greater in studies conducting in institution-based setting (25.78%) than community-based setting (15.50%), although the difference between the groups was not statistically significant (p = 0.56). (See Table 2).

Finally, when restricting our analysis by NOS quality score, the prevalence of AND was greater in low quality score studies (NOS score less than or equal to 8) studies (25.40%) than high quality score studies (NOS score above 8) (16.50%), although the difference between the groups was not statistically significant (p = 0.051). (See Table 2).

### Narrative review

#### Determinants of AND

In this section, we qualitatively analyzed the factors that were associated with an increased risk of AND in Ethiopia form the included five studies [32–36] **(Table 4**). In general, we observed that the level of adjustment for the possible confounding factors that are responsible for a greater risk of AND was inconsistent in Ethiopian studies. For example, in our review, we found that the association between the previous history of depression and AND was assessed only by two studies [32, 35]. Similarly, only two studies measured the link between AND and unplanned pregnancy[32, 36], and being house wife’s[33, 34]. Moreover, the association between AND and other factors was assessed inconsistently (i.e. in each study different types of factors are measured as possible predisposing factors for AND). Therefore, it was difficult to combine and provide the pooled mean effects of factors associated with AND in Ethiopia.

**Table 4:** Characteristics of factors associated with antenatal depression in Ethiopia by their odds ratio, confidence interval strength of association, author and year.

Factors	Odds ratio(AOR)	95% Confidence interval	Strength of association	Author, year
History of still birth	3.97	1.67–9.41	Strong, positive	Bisetegn, 2016
Complications during pregnancy	3.29	1.66–6.53	Strong., positive	Bisetegn, 2016
Previous history of depression	3.48	1.71–7.06	Strong, positive	Bisetegn, 2016
Having debt	2.79	1.33–5.85	Moderate, positive	Bisetegn, 2016
Unplanned pregnancy	2.39	1.20–4.76	Moderate, positive	Bisetegn, 2016
History of abortion	2.57	1.005–6.61	Moderate, positive	Bisetegn, 2016
3rd trimester of pregnancy	1.70	1.07–2.72	Moderate, positive	Bisetegn, 2016
Stressful life event	2.04	1.01–4.11	Moderate, positive	Bisetegn, 2016
House wife	2.57	1.21–5.46	Moderate, positive	Ayele, 2016
No ANC follow up	11.98	4.73–30,33	Strong, positive	Ayele, 2016
Irregular ANC follow up	11.43	3.68–35.49	Strong, positive	Ayele, 2016
Not satisfied by ANC follow up	4.78	1.71–13.11	Strong, positive	Ayele, 2016
Age group 20–29	0.18	0.07–0.49	Strong, negative	Ayele, 2016
Unmarried	4.07	1.18–14.04	Strong, positive	Mossie, 2017
Monthly income level<1500 Ethiopian birr	5.12	1.42	Strong, positive	Mossie, 2017
House wife	4.24	1.38–13.03	Strong, positive	Mossie, 2017
Previous history of depression	2.569	1.475–4.475	Moderate, positive	Biratu, 2015
Poor baby's father support	1.89	1.064–3.358	Moderate, positive	Biratu, 2015
Unplanned pregnancy	2.779	1.594–4.846	Moderate, positive	Biratu, 2015
Partner violence during pregnancy	3.41	1.18–9.10	Strong, positive	Dibaba, 2013
Food insecurity	4.60	2.75–7.70	Strong, positive	Dibaba, 2013
Farmer	3.43	1.95–6.05	Strong, positive	Dibaba, 2013
High social support	0.23	0.11–0.47	Strong, negative	Dibaba, 2013
Medium social support	0.27	0.14–0.53	Strong, negative	Dibaba, 2013
Unwanted pregnancy	1.96	1.04–3.69	Moderate, positive	Dibaba, 2013

In this study, the determinants of AND were categorized into the following three domains: sociodemographic factors (eight determinants), clinical and pregnancy-related factors (six determinants), and psychosocial and other factors (nine determinants). The overview of these factors including, the measures of effects, the strength of association and corresponding articles were presented in Table 4.

#### Sociodemographic factors

As summarized in Table 2, eight sociodemographic factors were significantly associated with an increased risk of AND [32–36]. The sociodemographic factors significantly associated with AND were low income (AOR = 5.12 CI;1.42, 18.48) [34], being unmarried (AOR = 4.07 CI;1.18, 14.04)[34], farmers (AOR = 3.43 CI;1.96–6.05) [36], and had debt (AOR = 2.79 CI;1.33,5.85). Furthermore, those who were housewife (AOR 2.57; CI;1.21;5.46) [33], had food insecurity (AOR = 4.60, CI; 2.75; 7.70) and employed (AOR = 2.50, CI; 1.13–5.56), and were in the age range 20–29 82 (AOR 0.18; CI; 0.07–0.49)[33] were associated with a higher risk of developing AND[36].

#### Clinical and pregnancy-related factors

As illustrated in Table 4, six clinical and pregnancy-related factors were positively and significantly associated with a higher risk of AND in Ethiopia [32–36]. Those pregnant women who were nullipara (AOR = 4.74, CI;1.58;14.17 [33], and had an unwanted pregnancy AOR = 1.96, CI;1.04–3.69)[36] were at a greater risk of developing AND.

Similarity, pregnant women who were in the third trimester of pregnancy (AOR = 1.70, CI (1.07, 2.72)) [32] and those whose had complications during pregnancy (AOR = 3.29 CI; 1.66, 6.53) [32]) linked with higher risk of developing AND.

In addition, in this review those whose pregnancy was unplanned [35] and had the previous history of depression [32, 35] were associated with a higher risk of developing AND.

#### Psychosocial and other factors

As indicated in Table 4, nine psychosocial and other factors were linked with increased risk of AND in Ethiopia. Those pregnant women who experienced partner violence during pregnancy [36], had lack of baby’s father support during pregnancy) [35], had risky stressor [33] and those not satisfied by ANC follow-up) [33] were linked with a considerably higher risk of developing AND. Similarity, those who had medium social and high social support were less likely to have AND as compared to those who have low social support [36].

Moreover, those who have a history of abortion and stillbirth,[32], had irregular ANC follow-up pattern and no ANC follow up were more likely to have AND [33].

## Discussion

In this systematic review and meta-analysis, we explored the prevalence and determinants of AND in Ethiopia. Five cross-sectional studies were included in the final analysis. Based on the meta-analysis a significant proportion (more than 1 in 5) of women had AND in Ethiopia. This shows that AND is a significant public health problem in Ethiopia. We also identified 23 determinants that were significantly associated with antenatal depression in Ethiopia.

In this study, the pooled prevalence of AND in Ethiopia was 21.28% (95% CI 15.96–27.78). These findings were consistent with the results from a recent meta-analysis that found pooled estimates for AND in the low and middle-income countries (LMICs) 25.3% [11]. However, our results were lower than unpooled results from the studies conducted in some low and middle-income countries (LMICs), such as, 29 % in Bangladesh [28], 39 % in South Africa Cape Town, 38.5 %[29] in South Africa KwaZulu-Natal[30] and 39.5 %, in Tanzania [31]. The results of this meta-analysis were higher than in studies conducted in other low and middle-income countries (LMICs), such as 20.2 % in Brazil [26], 25 % in Pakistan[27]. These differences might be due to the socioeconomic and cultural differences between the countries. The other possible reasons for the observed variation may be the use of a different instrument to assess antenatal depression. Moreover, the other obvious reason for the various might be the sample size, a collection of data from different settings (community and institution setting) as well as different study periods. This is because in the above studies the findings were based on the single study, but our findings were pooled prevalence estimates based on the five studies conducted in different setting and periods.

We also measured the pooled prevalence estimate of AND in each trimester of pregnancy. Our results found that the pooled prevalence estimate of AND was 19.13% (4.16%, 56.31%) in the first trimester of pregnancy. This finding was in line with a meta-analysis study done in developing countries which reported the pooled prevalence of AND 7.4% in the first trimester of pregnancy. In the current study, the pooled prevalence estimates of AND were 18.86% (11.51%, 28.87%) in the second trimester and 32.10% (12.93%, 60.07%) in the third trimester of pregnancy. These study findings are higher than a meta-analysis study done in developing countries which showed the pooled prevalence of AND 12.8% and 12.0% 16.7% for the second, and third trimesters respectively[1]. These differences might be due to the sample size, socioeconomic and cultural differences between the countries.

In the current review, a subgroup analysis of the prevalence of AND by the instrument used showed that pooled prevalence result of AND of studies conducted by using beck depression inventory (BDI) was significantly higher in the results of the studies done using EPDS. The pooled prevalence of AND was 26.48% (95%CI;19.09%, 35.48%) for the studies done by using beck depression inventory (BDI) and the pooled prevalence of AND was 18.28% (95% CI; 12.13–26.60) for the studies done by using EPDS. This difference might be due to BDI was not specifically prepared for detecting depression in pregnant population and it is also not validated in Ethiopia. Whereas EPDS is originally designed for assessing depression in pregnant and postpartum women and validated in Ethiopia. In addition, BDI has items used to assess somatic symptoms for depression which are usually common in pregnant women.

Subgroup analysis of the prevalence of AND by study setting, show that pooled prevalence result of AND of studies done in institution-based setting was significantly higher in the results of the studies done in the community setting. The pooled prevalence of AND was 15.50% (9.s%CI, 9.05–25.27) for the studies done in institution-based setting and the pooled prevalence of AND was 25.77% (95% CI 21.62–30.41) for the studies done in the community setting. This difference might be due to those who came to institution most likely have somatic symptoms and serious symptoms which are easily detected.

In our review we identified that factors which had robust scientific evidence as determining factor for AND in different worldwide studies, such as family history of depression, previous history of depression, substance use during pregnancy, any mental disorder during pregnancy, social support, unwanted pregnancy, stigma, partner violence during pregnancy, early pregnancy, stressful life events, complications during pregnancy, and economic status were either not included in the model or no assessed as confounding factors in each of the five included studies. Additionally, we observed that the level of adjustment for the possible confounding factors that are responsible for a greater risk of antennal depression was inconsistent in Ethiopian studies. For example, in our review, we found that the association between the previous history of depression and antenatal depression was assessed only by two studies [32, 35]. Similarly, only two studies measured the link between antenatal depression and unplanned pregnancy[32, 36], and being house wife’s [33, 34]. Moreover, the association between AND and other factors was assessed inconsistently (i.e. in each study different types of factors are measured as possible predisposing factors for antenatal depression). Therefore, the association observed between AND and various factors reported in those included studies might be due to an inadequate level of adjustment for the possible confounding factors.

### Difference between the studies included in systematic review and meta-analysis

The variations between the included studies resulted in an apparent heterogeneity in our meta-analysis. The instrument used to measure AND, the stage of pregnancy, the study setting, the sample size, and the study population varied on several characteristics which might have contributed for the observed difference in the magnitude of AND among the studies in Ethiopia. To further identify the possible source of heterogeneity in the estimates of AND, we conducted a leave-one-out analysis. The analysis showed that our findings were robust and not dependent on a single study.

In our sensitivity analysis, we found that the main cause of the observed variation in the prevalence of AND was found to be the instrument used to estimate AND. The prevalence was 26.8% as measured by BDI and 18.82% as measured by EPDS with significant p-value between the studies conducted using the above two instruments (p value = 0.032). The possible explanation for the remarkable difference in the prevalence of AND among the various measuring instrument (BDI and EPDS) might be due to variations in the sensitivity (the test's ability to correctly identify a participant with the disease as positive) and specificity (the test's ability to correctly label a participant without the disease as negative) of the instruments. Epidemiologic evidence showed that the use of BDI during pregnancy was associated with an apparent overestimation of the magnitude of depression during pregnancy due to the existing overlap between depressive and somatic symptoms during pregnancy [44]. Therefore, a considerably higher magnitude of AND in studies conducted using BDI than EPDS may be due to the overlap between BDI and somatic symptoms of pregnancy. The results of our study support the view that validation and use of the standard instrument for screening and diagnosis of depression during pregnancy.

However, the difference observed among the included studies depending on the trimesters of pregnancy, the study setting, as well as the quality score, was not significant. Nevertheless, the sample size and a small number of studies included in each subgroup in our sensitivity analysis need to be acknowledged.

Finally, in order to make the findings of our meta-analysis meaningful, we employed a random-effects model. It’s widely held that the summary effect estimates in the random effect model are more conservative than fixed-effects summaries in epidemiologic meta-analysis.

### Strength and limitations

This study has several strengths: First, we used a prespecified protocol for search strategy and data abstraction and conducted quality assessment two independent investigators to lessen the possible assessor bias; Second, we employed subgroup and sensitivity analysis based on study setting, instrument used, and trimester of pregnancy to identify the small study effect and the risk of heterogeneity in; third, The quality of included studies was evaluated by two authors. Nevertheless, our systematic review and meta-analysis has some limitations: first, in our small number of studies were included in our subgroup analysis which reduce the precision of the estimate; second, a considerable heterogeneity was identified among the studies; due to inconsistent adjustment and inclusion of factors determining antenatal depression we did only qualitative analysis for associated factors of antenatal depression.

## Conclusion and recommendations

In summary, in our meta-analysis, the pooled prevalence of AND in Ethiopia was 21.28% (95% CI; 15.96–27.78). The pooled prevalence of AND was highest in the third trimester of pregnancy at 32.10% and it was 19.13% in the first trimester and 18.86% in the second trimester of pregnancy. In addition, the pooled prevalence of AND was 26.48% and 18.28% as measured by BDI and the EPDS, respectively. The pooled prevalence of AND was 15.50% for the studies conducted in the community setting and it was 25.78% for the studies conducted in the institution-based setting.

In this study, we observed that the level of adjustment for the possible confounding factors that are responsible for a greater risk of AND was inconsistent in Ethiopian studies.

Studies on the magnitude of antenatal depression and determinants in each trimester of pregnancy with representative sample size and studies focusing on scientifically plausible and consistent factors on AND are recommended.

Future epidemiologic studies focusing on specific age groups such as adolescents, young adults, and disadvantaged women as well as on women with unintentional pregnancy, experienced violence and those who are substance and drug users during pregnancy were warranted. Furthermore, longitudinal studies focusing on incidence and determinates of AND are recommended.

Attention needs to be given for the mental health of pregnant women and the government needs to consider the possible integration of mental health services with the existing antenatal care in Ethiopia.

Finally, future studies have to be conducted using a validated instrument to be used in pregnant women.

## Supporting information

S1 Table PRISMA-P (Preferred Reporting Items for Systematic review and Meta-Analysis Protocols) 2015 checklist: Recommended items addressed in our systematic review and meta-analysis. (DOC)

S2 Table Summary of quality assessment and agreed level of bias and level of agreement on the methodological qualities of included studies in meta-analysis based on sampling, outcome, response rate and method of analysis. (DOCX)
