# Supplementary material for: Prevalence and determinants of antenatal depression in Ethiopia: A systematic review and meta-analysis
Source: PLoS One. 2019 Feb 19;14(2):e0211764. doi: 10.1371/journal.pone.0211764 (PMC6380851; doi:10.1371/journal.pone.0211764)
Supplement: S2 Table — (DOCX) [file pone.0211764.s002.docx]

**S2 Table:**  Summary of the quality and an agreed level of bias and level of agreement on the methodological qualities of included studies in a meta-analysis based on sampling, outcome, response rate and method of analysis

| Study | Overall agreement and precision | | | NOS Scale quality score (from a total 9-point score) |
| --- | --- | --- | --- | --- |
|  | Percentage of agreement | Kappa value | Level of agreement |  |
| Bisetegn TA. et.al (2016) [29] | 100 | 1 | almost perfect | 9 |
| Ayele TA et. al. (2016) (30) | 75 | 0.56 | Moderate | 8 |
| Biratu, A.,et. al (2015) (31) | 100 | 1 | Almost perfect | 8 |
| Dibaba Y et.al (2013) (33) | 100 | 1 | Almost perfect | 9 |
| Mossie TB.et.al (2015) (32) | 75 | 50 | Moderate | 8 |
